# Evaluation of Excipient Risk in BCS Class I and III Biowaivers

**DOI:** 10.1208/s12248-021-00670-1

**Published:** 2022-01-05

**Authors:** Melissa Metry, James E. Polli

**Affiliations:** Department of Pharmaceutical Sciences, School of Pharmacy, University of Maryland, Baltimore, Maryland USA

**Keywords:** bioavailability, bioequivalence, Biopharmaceutics Classification System (BCS), biowaiver, excipient

## Abstract

The objective of this review article is to summarize literature data pertinent to potential excipient effects on intestinal drug permeability and transit. Despite the use of excipients in drug products for decades, considerable research efforts have been directed towards evaluating their potential effects on drug bioavailability. Potential excipient concerns stem from drug formulation changes (e.g., scale-up and post-approval changes, development of a new generic product). Regulatory agencies have established *in vivo* bioequivalence standards and, as a result, may waive the *in vivo* requirement, known as a biowaiver, for some oral products. Biowaiver acceptance criteria are based on the *in vitro* characterization of the drug substance and drug product using the Biopharmaceutics Classification System (BCS). Various regulatory guidance documents have been issued regarding BCS-based biowaivers, such that the current FDA guidance is more restrictive than prior guidance, specifically about excipient risk. In particular, sugar alcohols have been identified as potential absorption-modifying excipients. These biowaivers and excipient risks are discussed here.

**Figure:**
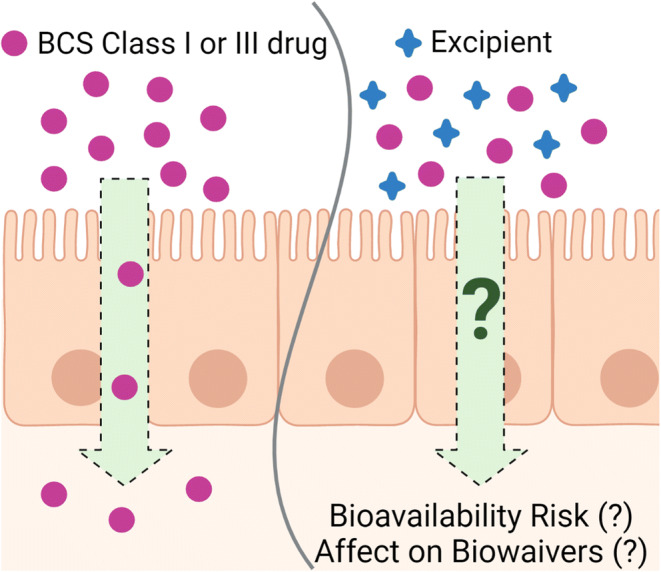
Graphical Abstract

Graphical Abstract

## INTRODUCTION

Following oral administration, solid dosage formulations must first disintegrate in the gastrointestinal (GI) tract and dissolve in solution for drug absorption to occur. Based on the drug’s physiochemical properties, intestinal permeation occurs by passive diffusion or active/facilitative transport (1). Of the fraction of the oral dose that is absorbed from the intestinal lumen, the fraction that becomes available in systemic circulation (i.e., is bioavailable) is further reduced by metabolism through the gut wall into the hepatic portal circulation, metabolism by first pass elimination through the liver, and biliary excretion (2).

Oral drug absorption is a process that is influenced by key biopharmaceutical and physiological factors. Important physiochemical properties of the drug include its solubility, intrinsic dissolution rate, ionization (pK_a_), lipophilicity (log P), stability, surface area, crystallinity, polymorphism, salt form, and molecular size. Physiological factors such as gastrointestinal pH, gastric emptying, small intestinal transit time, bile salts, and mechanisms of membrane permeability also influence oral drug absorption (3). Non-drug components of the dosage formulation, i.e., excipients, may also impact absorption of the drug. Excipients are typically used in dosage formulations to ensure manufacturability and content uniformity but are also used to modulate drug substance or active drug ingredient (API) stability and bioavailability. Generally, excipients can potentially have an impact on drug absorption by altering the dosage formulation’s disintegration, dissolution, or stability, or by directly impacting GI physiological processes.

It is well appreciated that excipients can alter drug release rate and/or extent of release from dosage formulations. However, there are several anticipated mechanisms through which excipients in the GI tract could impact drug absorption. For example, excipients may potentially modify GI transit time and luminal volumes, alter permeability, or modify metabolism within the GI tract (4). Osmotically active excipients such as sugar-alcohols (e.g., mannitol, sorbitol) and polyethylene glycol (PEG) 400 are known to potentially reduce drug absorption by increasing GI fluid volume, which in turn dilutes intraluminal drug concentration and reduces small intestinal transit time (5). However, other potential concerns such as excipient impact on drug membrane permeability have much less evidence of an effect *in vivo* (5–9).

Given the potential excipient risks to drug absorption, changes to drug formulations should consider excipient amount, mechanism(s) in which excipient may impact absorption, and the drug’s absorption properties (10,11). *In vivo* bioequivalence (BE) studies are generally needed to demonstrate a lack of impact of significant formulation changes on a drug’s bioavailability during its development, for post-approval manufacturing changes, and when developing generic products. A regulatory framework to provide regulatory relief based on the *in vitro* characterization of the drug substance and drug product, termed Biopharmaceutics Classification System (BCS), allows the waiving of clinical BE studies for some immediate-release (IR) solid oral dosage formulations. Not needing human BE trials provides a great benefit in that it reduces drug development costs and eliminates unnecessary clinical trials (12). For various reasons, *in vitro* studies are sometimes better than conventional human pharmacokinetic *in vivo* studies in assessing BE of IR solid oral dosage formulations (13).

## BCS CLASSIFICATION AND BCS-BASED BIOWAIVERS

The BCS classifies orally administered immediate release drug products based on the fundamental principles that control the rate and extent of drug absorption, i.e., solubility, dissolution rate, and intestinal permeability. The categories are high solubility-high permeability (Class I), low solubility-high permeability (Class II), high solubility-low permeability (Class III), and low solubility-low permeability (Class IV) (14).

The first finalized US Food and Drug Administration (FDA) BCS guidance for industry was issued in August 2000 and indicated that evidence of BE *via in vitro* dissolution studies in lieu of *in vivo* pharmacokinetic profiles may be sufficient for BCS Class I drugs. Such biowaivers were also supported by the European Medicines Agency (EMA) in their guidance issued in 2001. EMA and FDA expanded BCS-based biowaivers to include Class III drugs in 2010 and 2017, respectively (15,16). Although the World Health Organization (WHO) considered granting BCS biowaivers for Class II weak acids, the organization published a guideline in 2015 for only Class I and Class III generic drugs (17).

The International Council for Harmonisation of Technical Requirements for Pharmaceuticals for Human Use (ICH), which involves experts from both regulatory and industry agencies, finalized a guideline intended to be recognized worldwide, entitled “M9 guideline on biopharmaceutics classification system-based biowaivers (Step 5)” in 2020 (10). With the support of ICH, FDA recently finalized a guidance for industry in May 2021 entitled “M9 Biopharmaceutics Classification System-Based Biowaivers,” which replaced the 2017 FDA guidance (11). Relative to the 2017 FDA guidance, the 2021 FDA guidance (i.e., M9 document) has some biowaiver acceptance criteria changes, as summarized in Table I. Both documents indicate that BCS Class I and III drug products may be eligible for a biowaiver for IR oral dosage formulations with the same strength as the reference product. Acceptance criteria consist of the composition (i.e., excipients) and *in vitro* dissolution performance of the drug product depending on its BCS classification (11). It should be noted that M9 is a notable step forward, as it is the first harmonized allowance of BCS-based regulatory relief, including for example, in Japan.

**Table I:** Comparison of 2017 FDA *Versus* 2021 M9 BCS-Based Biowaiver Criteria

Criteria	2017 FDA Guidance	2021 M9 Guidance
Dosage form	Solid oral dosage forms	Solid oral dosage forms or suspensions
Drug substance	Must be the same	Different salt form may be applicable (BCS Class I); ester, ether, isomer, mixture of isomers, complex or derivative are not applicable
Solubility class boundary or drug amount	Highest strength	Highest single therapeutic dose
Solubility assessment	Ionization determines number of pH conditions within 1–6.8, including pH=pKa; pH=pKa+1; pH=pKa-1, and at pH = 1 and 6.8	At least three pHs within 1.2–6.8, including buffers at pH 1.2, 4.5, and 6.8
Permeability assessment	Preference for human PK studies (e.g., absolute bioavailability or mass balance); *in vivo* human intestinal perfusion; animal *in vivo* or *in situ* intestinal perfusion, excised animal/human intestinal tissues, or epithelial cell monolayers possible for passively absorbed drugs, although human data supersedes	Preference for human PK studies (e.g., absolute bioavailability or mass balance); Caco-2 permeability is considered for passively absorbed drugs
Excipients	*BCS Class I:* Generally, excipients will not affect rate or extent of absorption; consider excessive quantities of surfactants (e.g., polysorbate 80) and sugar alcohols (e.g., mannitol or sorbitol) *BCS Class III:* Excipients must be qualitatively the same and quantitatively similar, except for excipients used in limited amounts such as the coating/shell	*BCS Class I:* Excipients that may affect absorption of the particular API are qualitatively the same and quantitatively similar (i.e., within ±10% of the weight of excipient in the reference product and a cumulative difference within ±10%); any qualitative and quantitative differences are acceptable for all other excipients *BCS Class III:* Excipients must be qualitatively the same and quantitatively similar, except for excipients used in limited amounts such as colorants/flavoring
*In vitro* dissolution performance	Demonstrate *f*_2_ similarity factor of ≥ 50; not necessary if test and reference both have very rapid properties (≥85% for the average percent dissolved in ≤ 15 min) To allow the use of mean data for *f*_2_, the coefficient of variation should not be more than 20% at the earlier time points (e.g., 15 min), and should not be more than 10% at other time points	*BCS Class I:* test and reference should both have, - very rapid properties (≥85% for the average percent dissolved in ≤ 15 min), or - rapid properties (≥85% for the average percent dissolved in ≤ 30 min) and *f*_2_ similarity factor of ≥ 50 If one product has rapid and the other has very rapid characteristics, demonstrate *f*_2_ similarity factor of ≥ 50 When the coefficient of variation is too high, *f*_2_ calculation is considered inaccurate and a conclusion on similarity in dissolution cannot be made *BCS Class III:* test and reference should both produce very rapid properties (≥85% for the average percent dissolved in ≤15 min)

However, M9 guidance is more restrictive than the prior 2017 FDA guidance, specifically about excipient risk. The 2017 FDA guidance indicated, in the context of BCS-based biowaivers, “In general, using excipients that are currently in FDA-approved IR solid oral dosage forms will not affect the rate or extent of absorption of a highly soluble and highly permeable drug substance that is formulated in a rapidly dissolving IR product.” On the other hand, M9 lacks such a statement. The prior 2017 FDA guidance also indicates, “Unlike for BCS class 1 products, for a biowaiver to be scientifically justified, BCS class 3 test drug product must contain the same excipients as the reference product,” and further describes evaluation of “the same excipients” (e.g., qualitatively the same and quantitatively similar). While the prior 2017 FDA guidance anticipates common excipients to not be a concern for BCS Class I, this expectation is less evident from the M9 guidance. M9 does indicate “[BCS Class I drugs] generally represent a low risk group of compounds in terms of the potential for excipients to affect absorption, compared to other BCS classes. Consideration of excipient effects for BCS Class I drug products should focus on potential changes in the rate or extent of absorption.” However, this statement is only relative to other BCS classes and apparently does not convey the anticipation that common excipients are not a concern for BCS Class I drugs. An additional restriction is observed where Caco-2 is indicated as the only *in vitro* permeability assessment in M9, while the prior guidance states *in vitro* permeability methods using excised intestinal tissues, or monolayers of suitable epithelial cells, may be used. This difference is a notable narrowing.

EMA and FDA have published product-specific guidances, and the International Pharmaceutical Federation (FIP) has published over 50 drug monographs that assess potential usage of BCS biowaivers (17). BCS-based biowaiver monographs are a series of literature reviews on IR solid oral dosage formulations published in the *Journal of Pharmaceutical Sciences*. Evaluation of the API’s physiochemical properties, pharmacokinetics, and interactions with excipients are considered for biowaiver risk-based analysis. Many of the monographs support biowaivers for specific drugs and their corresponding IR dosage formulations, such as metformin, sitagliptin, and moxifloxacin (18–20). Meanwhile, a smaller number of monographs suggest against biowaivers for specific drugs and their corresponding IR dosage formulations, such as carbamazepine (i.e., due to its narrow therapeutic index) (21).

It should be noted that when comparing BE *in vitro* test results to *in vivo* test results, *in vivo* BE studies can have type I (i.e., consumer risk/false positive) and type II (i.e., producer risk/false negative) errors. Hence, a reason for discordance between a BCS biowaiver conclusion and an *in vivo* BE conclusion is type II error in *C*_max_ from *in vivo* pharmacokinetic (PK) BE studies. Of the *in vivo* BE metrics for rate and extent, *C*_max_ is the more common reason for BE failure (22–25). Limitations of *C*_max_ as a BE metric are well described (26–29). In a retrospective study performed in Brazil, 12 of 115 studies of Class III drug products provided non-bioequivalent (i.e., non-BE, where confidence interval exceeds 80–125% range) result, with 5 of those being bioinequivalent (i.e., point estimate is outside the range of 80–125%) (30). Specifically, among the 12 non-BE studies, 7 were due to only *C*_max_, 4 were due to both *C*_max_ and AUC_0-*t*_, and 1 was due to only AUC_0-*t*_. Of the 5 bioinequivalent studies, 4 were caused by only *C*_max_ and 1 was due to *C*_max_ and AUC_0-*t*_. Similarly, for Class I drug products, 22 out of 140 studies provided a non-BE result, with 8 of those being bioinequivalent. Of the 22 non-BE studies, 18 were due to only *C*_max_ while 4 were due to both *C*_max_ and AUC_0-*t*_. Of the 8 bioinequivalent results, 7 were due to only *C*_max_ while 1 was due to both *C*_max_ and AUC_0-*t*_. Thus, it is important to consider that *in vivo* bioinequivalence can be due to limitations of *C*_max_.

## CLASSIFICATION OF BCS CLASS I AND III AND ELIGIBLITY FOR BIOWAIVER

According to the current FDA guidance, to be considered highly soluble, the highest single therapeutic dose must be soluble in 250 mL or less of aqueous media in at least three pHs within the range of 1.2–6.8 at 37±1°C. To be considered highly permeable, the drug product must have human pharmacokinetic results with an absolute bioavailability of ≥85% or a urine recovery of ≥85% *via* mass balance with demonstrated GI stability. Other *in vivo* data such as drug recovery in feces or data obtained from the literature (e.g., product knowledge and bioavailability studies) may be acceptable. *In vitro* methods include Caco-2 cell permeability assays, but they should be used alongside available *in vivo* data to estimate intestinal drug absorption. If Caco-2 permeability assays are used alone to classify a drug as highly permeable, classification is limited to passively absorbed drugs due to the lack of certain intestinal transporters in the Caco-2 cell model (11).

Using comparative *in vitro* dissolution tests, the test and reference products of BCS Class I drugs must dissolve very rapidly (≥85% for the mean percent dissolved in ≤15 min), or rapidly (≥85% for the mean percent dissolved in ≤30 min) with similar *f*_2_ (≥50) comparison. BCS Class III drugs must demonstrate very rapid comparative *in vitro* dissolution (11). Interestingly, the 2021 FDA Guidance does not allow for other methods to compare *in vitro* dissolution profiles other than *f*_2_.

## POTENTIAL ABSORTION-MODIFYING EXCIPIENTS

FDA’s Inactive Ingredient Database (IID) catalogs all excipients used in approved New Drug Application (NDA) and Abbreviated New Drug Application (ANDA) products, regardless of current market availability of the drug product (31). Information on each excipient includes ingredient name, route of administration, dosage formulation, chemical abstracts service (CAS) number, unique ingredient identifier (UNII), maximum potency per unit dose, and maximum daily exposure (MDE).

Excipients are usually in much greater amounts than the API and can typically make up to ~90% of the entire drug product. They are used in dosage forms to facilitate manufacturability, stability of the API, dose uniformity, and delivery of the API to the systemic circulation. Commonly used excipients can be grouped into several categories based on their functions such as binders (e.g., hypromellose, starch, povidone), fillers (e.g., lactose, mannitol), lubricants (e.g., magnesium stearate, stearic acid), and surfactants (e.g., sodium lauryl sulfate, polysorbates). Excipients are generally considered as “inactive ingredients.” Some excipients (e.g., polymers) are utilized as enhancers of solubility or dissolution rate for poorly soluble drugs.

It can be conceived that absorption-modifying excipients (AMEs) that do not affect *in vitro* dissolution testing can be considered “critical” (i.e., concerning) for biowaivers since their effect on GI absorption (e.g., transit, intestinal permeability) would be overlooked. In the literature, only a subset of mechanisms exist by which excipients could function as AMEs and potentially affect the absorption of BCS Class III drugs. In contrast, BCS Class I drug absorption is not likely to be impacted by such excipients. For many potential AMEs, there is no *in vivo* evidence of such an effect. For example, we know of no common excipient that decreased drug absorption of a BCS Class I drug by drug-excipient binding where *in vitro* testing did not anticipate such binding. Many of the excipients with such reported effects, such as surfactants, would not normally be used in IR solid oral dosage forms for highly soluble drugs, i.e., BCS Class I and III. Only a few potential AMEs have been identified, such as excipients that can impact intestinal transit (e.g., sorbitol, mannitol). Evidence of an effect in humans for several of these AMEs has largely been observed with only high quantities of excipient (e.g., 1.6 g of sorbitol) (9). Although surfactants have also been associated as potentially critical AMEs, there is no clear evidence in humans. Rather, evidence has been observed in the preclinical and *in vitro* domains.

### Sorbitol and Mannitol

The sugar alcohols sorbitol and mannitol are known to generate, in sufficient doses, significant osmotic effect after oral administration (i.e., decreases transit time that reduces drug absorption, particularly for low permeability drugs). The molecular weights of sorbitol and mannitol are the same (i.e., 182 Da), as they are stereoisomers, and they exhibit a dose-dependent proportional decrease in small intestinal transit time (32,33). It is important to consider the amount of excipient used, the degree at which that amount affects BCS Class I or III drug absorption, and the absorption properties of the drug substance such as the location, rate, and mechanism (4,11).

Chen *et al.* showed BCS Class III drugs were more sensitive than BCS Class I drugs to sorbitol, with respect to the ability of 5 g sorbitol to reduce drug absorption. Chen *et al.* employed metoprolol and ranitidine as Class I and Class III drugs, respectively. Test product containing 5 g of sorbitol had no impact on metoprolol extent of absorption compared to reference product containing sucrose in healthy volunteers. Meanwhile, ranitidine absorption was reduced by 7%, 25%, and 45% by 1.25 g, 2.5 g, and 5 g sorbitol, respectively (6). In general agreement with Chen *et al.’s* observation regarding metoprolol, Fassihi *et al.* observed that 10 g of sorbitol had no impact on the extent of theophylline, a BCS Class I drug (7,8).

Vaithianathan *et al.* assessed commercial solutions containing sorbitol of each cimetidine and acyclovir, BCS Class III drugs, in bioequivalence studies (9). The cimetidine and acyclovir solutions contained about 1.6 g and 1.5 g of sorbitol, respectively. The dose containing 1.6 g sorbitol reduced cimetidine absorption by 19%. Conversely, 1.5 g sorbitol did not impact acyclovir absorption. It is not clear why these similar doses of sorbitol showed apparently different impacts, although cimetidine and acyclovir are of course different drugs, including with different formulation compositions. Vaithianathan *et al.* also compared the cimetidine commercial solution (with about 1.6 g of sorbitol) against a sorbitol-free solution of cimetidine (9). While *C*_max_ was reduced by about 13%, cimetidine absorption was not impacted. Overall results support Chen *et al.’s* observation that 1.25 g sorbitol can impact Class III drug absorption. An *in silico* model conducted by Yamane *et al.* estimated that a threshold of 400 mg of sugar alcohols will not impact drug absorption (34).

Other studies examined quantities more than 2 g of sugar alcohols. Adkin *et al.* evaluated the impact of 2.264 g of mannitol on cimetidine absorption, as well as small intestinal transit times. Mannitol reduced cimetidine absorption by about 31%, as well as small intestinal transit time by 23% (35). Also, Adkison *et al.* observed that 3.2, 10.2, and 13.4 g of sorbitol decreased absorption of lamivudine, a BCS Class III drug, by 20%, 39%, and 44%, respectively (36,37).

### Surfactants

Surfactant effect on permeability has been studied *in vivo* and *in vitro*, in regard to reducing small intestinal transit time or modifying passive or active permeation (4). However, *in vivo* human data has shown no effect.

The surfactant sodium lauryl sulfate (SLS) has been shown to increase permeability of mannitol and other drugs in Caco-2 monolayers *via* opening of tight junctions (38). SLS has also been classified as a modulator of paracellular transport from *ex vivo* data (39). Parr *et al.* showed SLS at ≥ 0.1 mg/mL increased permeability across Caco-2 monolayers of four BCS Class III drugs due to damaging membrane integrity, but not the BCS Class I compound antipyrine. Concentrations of 0.01–0.04 mg/mL did not have any effect on the permeability of all five drugs (40). Although García-Arieta considered the *in vivo* impact of SLS where two studies showed bioinequivalence (with 3.64 mg or 1.5 mg SLS), other studies with very high amounts (9 g) have demonstrated bioequivalence (5). Vaithianathan *et al.* found that sodium lauryl sulfate (25 mg) had no significant impact on the *in vivo* bioavailabilities of cimetidine nor acyclovir (9).

The surfactants Vit-E-PEG, AOT, polysorbate 80, CTAB, polysorbate 20, Cremophor® EL, Solutol® HS 15, and Brij® 58 and the polymer NaCMC have been shown to be inhibitors of P-glycoprotein (P-gp) in MDCK-MDR1 cell culture, as determined by significant intracellular increase in digoxin (41). In an *in vivo* rat experiment, surfactants modified the pharmacokinetic profile of orally administered digoxin and celiprolol (BCS Class III), although the overall AUC was not increased. An early peak of absorption was observed consistently across surfactants, most likely due to the higher concentration of excipient in the proximal intestine where P-gp expression is lower (42).

## REGULATORY FRAMEWORK IN FORMULATION

Biowaivers are allowed for scale-up and post-approval changes (SUPAC) to drug formulations. The FDA guidance on “Immediate Release Solid Oral Dosage Forms Scale-Up and Post-Approval Changes” (SUPAC-IR) published in November 1995 outlines post-approval changes in the composition of formulation, manufacturing location, batch size, and manufacturing equipment and process. SUPAC-IR provides regulatory relief in the context of BCS. Specifically, excipient changes are divided into three impact levels on formulation quality and performance that are accepted by dissolution and *in vivo* BE requirements. The categories include level 1 (negligible impact), level 2 (could have a significant impact), and level 3 (likely to have a significant impact) (43).

Changes in excipients at level 1 are unlikely to affect the quality or performance of the formulation such as in the color or flavor of the drug product or excipient amounts less than or equal to a percentage (w/w) of the total formulation. Specifically, ±5% for fillers, ±3% for the disintegrant starch and ±1% for other disintegrants, ± 0.5% for binders, ±0.25% for the lubricants calcium or magnesium stearate or ±1% for other lubricants, ±1% for talc and ±0.1% for other glidants, and ±1% for film coating. Additionally, the total additive excipient changes must not be >5%. BE is demonstrated in level 1 *via in vitro* dissolution testing (i.e., biowaiver) (43).

Changes in excipients at level 2 could significantly alter the quality or performance of the formulation. Examples include a change in the technical grade of an excipient or in the percent (w/w) of the total formulation greater than level 1 but less than or equal to a two-fold increase over level 1 changes. Additionally, the total additive excipient changes must not be >10%. BE for level 2 is demonstrated *via* dissolution profile similarity factor *f*_2_ (i.e., biowaiver) for BCS Class I, II, and III, with an exemption for BCS Class I drugs that show ≥85% dissolution in 900 mL 0.1N HCl in 15 min.^43^

Changes in excipients at level 3 significantly alter the quality or performance of the formulation due to additive excipient changes of >10% and require *in vivo* BE testing for qualification (43,44).

FDA expanded its SUPAC-IR requirements in the guidance for ANDAs, in which excipient changes must be “Q1/Q2,” i.e., the test formulation must be the same excipients (qualitatively the same; Q1) and in the same concentration (quantitively the same; Q2) to the reference formulation (45). Allowable qualitative excipient differences to be Q1 include those that affect the color or flavor of the drug product, printing ink, technical grade and/or specification, and particle size. Allowable quantifiable excipient differences to be Q2 include excipient amounts less than or equal to a percentage (w/w) of the total formulation. Specifically, the guidance for ANDAs states ≤10% for fillers, ≤6% for starch and ≤2% for other disintegrants, ≤3% for binders, ≤0.5% for the lubricants calcium or magnesium stearate or ≤2% for other lubricants, ≤2% for the glidant talc or ≤0.2% for other glidants, and ≤2% for film coating. Additionally, the total additive excipient changes must be ≤10% (45).

## EXCIPIENT RISK FOR BCS CLASS I

BCS Class I drug products have minimal risk regarding excipient changes since they are very well absorbed given their high solubility and high permeability characteristics. Since the rate-determining steps are dissolution, permeation, or gastric emptying, excipients that could alter the rate or extent of the drug’s absorption should still be evaluated. Such cases include excipients that modulate uptake transporters that the drug relies on for its high permeability, or excipients that increase the absorption rate of drugs that are absorbed slowly. A biowaiver is acceptable for BCS Class I drugs if the excipients that may affect absorption are qualitatively the same (i.e., identical chemistry, grade, and characterization) and quantitatively similar (i.e., within ±10% of the weight of excipient in the reference product and a cumulative difference within ±10%). For all other excipients, any qualitative and quantitative differences in excipients are acceptable when granting a biowaiver (10,11).

Cephalexin, a BCS Class I drug, has high intestinal permeability due to active uptake across the apical membrane of enterocytes *via* the proton-coupled oligopeptide transporter PEPT1. Variations in the expression of PEPT1 *in vitro* and *in vivo* (i.e., Caco-2 cells, human duodenum, and rat jejunum) are correlated with differences in the permeability of cephalexin (46). Hypothetically, excipients that have the potential to modulate PEPT1 activity or expression could affect the extent of absorption of cephalexin and would be important to consider during formulation development. In general, a 10–15% change in extent of absorption can be expected to cause bioinequivalence (47).

*In vitro* data has shown the non-ionic surfactants Solutol® HS15 (poly-oxyethylene esters of 12-hydroxystearic acid), polysorbate 20, and polysorbate 80 inhibit PEPT1 in transfected MDCKII cells (48). Notably, surfactants are also known to enhance the penetration of drugs through the intestinal membrane by disrupting its integrity and function. Therefore, it is important to consider an overall net effect since multiple mechanisms may (or may not) be at play when surfactants are present in the GI tract (49). Similarly, *in vitro* and *in situ* experiments have shown the excipient caprylocaproyl macrogolglycerides to enhance cephalexin transport. However, these experiments fail to emulate the *in vivo* human environment that includes active transport and fail to consider an already high permeability of cephalexin (46).

## EXCIPIENT RISK FOR BCS CLASS III

BCS Class III drug products are thought to be at risk of excipient changes since they have low permeability and may only be locally absorbed at specific sites along the gastrointestinal tract (e.g., only small intestine as colonic permeability is too low). Therefore, according to the current FDA guidance, a biowaiver is acceptable for BCS Class III drugs if all excipients are qualitatively the same (i.e., identical chemistry, grade, and characterization) and quantitatively similar (i.e., within ±10% of the weight of excipient in the reference product and a cumulative difference within ±10%), except for excipients that are used in limited amounts such as the film coating or capsule shell. This criterion assumes that all excipients have the potential to affect absorption of the drug, regardless of known or suspected capability (10,11).

Osmotically active excipients at amounts used in formulations have been shown to alter the bioavailability of BCS Class III drugs (50). Sorbitol decreased ranitidine absorption by increasing intestinal fluid volume, and thus enhancing GI motility and decreasing ranitidine transit time. Mannitol decreased the bioavailability of cimetidine. PEG 400 accelerated transit time and altered the absorption of ranitidine (50).

Valacyclovir, a BCS Class III drug and prodrug of acyclovir, is more permeable than administration as the parent drug acyclovir due to active uptake of valacyclovir (but not acyclovir) *via* PEPT1 such that bioavailability is improved to >50% compared to 15% (9,51,52). Non-ionic surfactants have been shown *in vitro* to inhibit intestinal transporters, including *via* modulation of membrane fluidity (53–55). For example, polysorbate 80 has been shown to inhibit the intestinal transporter PEPT1 (48). Hypothetically, the PEPT1 substrate valacyclovir could be impacted by polysorbate 80, which may potentially decrease valacyclovir absorption, although FDA M9 regulatory guidelines do not describe methods to assess transporter-mediated excipient-drug interactions.

If inhibition of intestinal efflux transporters affects permeability, there could be a potential increase in bioavailability, although not concerning for passive permeability drugs. Cimetidine and famotidine, which are both BCS Class III drugs, are substrates for intestinal efflux mediated by P-gp. Concentration-dependent decrease of the secretion of both drugs *in situ via* single-pass intestinal perfusion studies in rats was obtained by P-gp inhibitors. Notably, the *in vivo* permeability of both drugs along the small intestine correlated with P-gp expression levels, thereby exhibiting segmental dependent intestinal absorption. Site-specific P-gp inhibition along the intestine, as observed by verapamil in the literature, may impact overall drug absorption (56). Many surfactants and one polymer have also been shown *in vitro* to inhibit P-gp in MDCK-MDR1 cells while five dyes and one suspending agent showed minimal inhibition in HEK293 cells (41,57).

The surfactant vitamin E TPGS (d-α-tocopheryl polyethylene glycol 1000 succinate) has been classified as an inhibitor of P-gp-mediated drug transport in Caco-2 monolayers and other cell lines (58,59). It has also been shown to enhance the oral bioavailability of the BCS Class III drug colchicine in rats (60). Notably, *in vitro* findings involving intestinal absorption of P-gp substrates have been performed using Caco-2 monolayers, although these cells have variable P-gp expression based on the culture conditions (61) and it is indicated that they overexpress P-gp (62).

Rege *et al.* assessed the influence of nine excipients (lactose, SLS, polysorbate 80, HPMC, docusate sodium, EDTA, propylene glycol, PEG 400, and anhydrous cherry flavor) on the Caco-2 permeability of seven low permeability compounds. Polysorbate 80 significantly increased apical-to-basolateral permeability of low permeability compounds *via* inhibition of active efflux as assessed by the lack of effect on mannitol permeability. SLS moderately increased drug permeability and affected Caco-2 monolayer integrity. The rest of the excipients showed minimal impact on the overall permeability of these compounds (54).

The surfactants salcaprozate sodium (SNAC) and sodium caprate (C_10_) are two of the most advanced AMEs that have gone through clinical testing for the oral delivery of macromolecules. Oral semaglutide, which has gone through multiple phase 3 clinical trials, is the first oral peptide therapeutic for type 2 diabetes in the form of a daily capsule. It is thought that SNAC promotes semaglutide absorption in the stomach by raising local pH to protect drug from degradation by gastric enzymes, as well as by inducing transcellular flux of semaglutide across the gastric epithelium of the stomach. C_10_ was assessed for use with oral insulin although dosage formulation development was discontinued. Low concentrations of C_10_ act *via* openings of tight junctions, while high concentrations *via* membrane perturbation (63).

## DISCUSSION

### Suitability of Experimental Models to Assess Drug Absorption

Intestinal absorption is often determined using *in situ* rat perfusion models or *in vitro* epithelial cell culture models. It is important to consider the utility of these alternative methods to drug absorption in humans. *In situ* rat perfusion models exhibit physiological differences from humans such as dilution, gastric emptying, degradation, and intestinal transit. However, this model favorably assesses drug transport in small intestinal tissue, the main *in vivo* absorption site. Notably, although rat and human tissue show similar drug absorption profiles, they exhibit distinct transporter and metabolic enzyme expression in the intestinal wall. Therefore, a rat model can be used to predict oral drug absorption in the small intestine of human, but not to predict oral bioavailability (64).

Caco-2 cell monolayers are a sensitive tool capable of distinguishing between high and low permeability values. However, they present practical limitations as an *in vitro* model to assess excipient effects on drug permeability. For example, a theoretical increase in drug intestinal permeability of a Class III drug by a theoretical absorption-modifying excipient from 60% absorption to 65% may or may not be detectable by Caco-2 monolayers, or by *in vivo* human bioequivalence testing. Since the Caco-2 permeability assay has an intraday variability of ~10% that is comparable to the bioequivalence similarity assessment (e.g., 10–15%), it would be difficult to reliably detect small excipient effects for low permeability drugs by Caco-2 monolayers. That is, a true enhancement of drug permeability of 10% across Caco-2 monolayers may not reliably be detected, even though a 10% increase in *in vivo* drug absorption may be important. In general, a 10–15% change in extent of absorption can be expected to cause bioinequivalence (47).

However, in spite of a lower limit of sensitivity of Caco-2 monolayers to drug permeability enhancement, in the literature, Caco-2 cells have been highly sensitive to excipient effects on drug permeability compared to *in vivo* (54). Consistent with the sensitivity of Caco-2 monolayers, excipients such as surfactants, disintegrants, and chitosans have shown an effect on drug permeability across Caco-2 monolayers but not *in vivo*. Caco-2 monolayers can be expected to frequently over-predict *in vivo* effect of excipients, such as SLS. This over-prediction is in part because Caco-2 monolayers do not secrete mucus, such that Caco-2 is much more sensitive to membrane disruptors (i.e., surfactants) than *in vivo*. Mucus creates a steric and interactive barrier against intestinal permeation such that its presence (or absence) may impact drug permeation (65). Therefore, the *in vivo* implication of an enhancement in *in vitro* Caco-2 permeability by an excipient is not clear.

A practically challenging topic is the comparison of permeability values (e.g., with and without excipient). There is currently no known universal method to assess permeability similarity when employing an *in vitro* model such as Caco-2 in assessing potential excipient effect. Although intraday variability in Caco-2 permeability is low, there is appreciable variability between studies (even conducted on the same day) that is often unexplained. This may lead statistical analysis *via t*-tests to false-positives, even before considering the high sensitivity of Caco-2 to predict *in vivo* human permeability. For example, Rege at al. reported a false-positive outcome in about 10% of all studies (54). These apparent effects were about 1.3-fold in magnitude, were not systematic, and attributed to variability effects than true excipients effects. Two studies were repeated and showed no subsequent excipient effect. In another two studies, “tighter” monolayers, as assessed by mannitol permeability, explained the decreased drug permeability. Parr *et al.* examined excipient effects on BCS Class III permeability using Caco-2 monolayers and rat perfusion (40). It was concluded that the four BCS Class III compounds would not be greatly impacted by the excipients. Permeability values were examined in the presence and absence of excipient, but no statistical tests were conducted. It would appear that permeability comparisons should not be limited to straight-forward *t*-tests (either with or without multiple comparisons correction).

Madin-Darby canine kidney (MDCK) cells have also been used in permeability assays since they favorably grow more rapidly than Caco-2 cells. In drug discovery/development interface programs that examine drug biopharmaceutic properties, MDCK monolayers are perhaps even more commonly used than Caco-2 monolayers. MDCK and Caco-2 cells both form into polarized epithelial monolayers. Although Caco-2 and MDCK cells differ biological in their source (human colon and canine kidney, respectively), their monolayers have comparable apparent permeability coefficient values. Notably, they each differ and vary in their own transporter expression levels (66–68).

Other cell culture models may be a more practical permeability model worth further evaluation, such as human colon carcinoma cell lines HT29-H and HT29-MTX, which form a monolayer with a mucosal barrier (69,70). Co-culture models of enterocyte-like Caco-2 cells with mucus-producing HT29-MTX cells have also been assessed for correlation with human *in vivo* studies, although relevant intestinal transporters are still not expressed (71–73). Bioengineering approaches that involve 3D co-cultures have also been reported in the literature as biomimetic models (74). Nonetheless, there is a need for novel *in vitro* models alongside the use of human and animal *in vivo* techniques to assess permeability (75).

### Considerations of Excipient-Transporter Interaction

M9 guidance requires a BCS-based biowaiver proposal to include a mechanistic and risk-based approach in assessing if differences between test and reference product (e.g. pre- and post-change SUPAC products, brand *versus* proposed generic) will not affect drug absorption. One such potential mechanism is transporter-mediated drug absorption. Although M9 guidance references important intestinal transporters, i.e., P-gp and BCRP, Caco-2 cell monolayers are limited in M9 to only assessing high permeability of passively transported drugs due to the potential lack of transporter expression. Transporter-mediated risk assessment of excipients is discussed. A critical question is “Are there excipients in the formulation with known or suspected effects on drug absorption?”

Regarding the potential for transporter-mediated excipient-drug interactions, two other FDA guidances are more comprehensive in assessing such risks than M9: “*In Vitro* Drug Interaction Studies — Cytochrome P450 Enzyme- and Transporter-Mediated Drug Interactions” and “Clinical Drug Interaction Studies — Cytochrome P450 Enzyme- and Transporter-Mediated Drug Interactions.” (76,77) These companion guidances are largely aimed at drug development in anticipating or assessing drug-drug interactions. The guidances are additionally supported with a website concerning tables that list substrates, inhibitors, and inducers of P450 enzymes and transporters (78). Nine transporters are discussed, including two apically localized efflux transporters that have significant expression in the intestine: P-gp and breast cancer resistance protein (BCRP). Given their location and directionality of transport, they have the potential to translocate drug back into the gut lumen and reduce drug absorption. Correspondingly, for a drug that is incompletely absorbed due to such efflux, an excipient that inhibits P-gp and/or BCRP has the potential to increase drug absorption. *In vitro* dissolution would presumably not detect such an excipient effect.

The quality of experimental tools to evaluate transporter-mediated drug interactions varies and depends upon the question to be addressed. There are *in vitro* assays that are viewed as reliable to demonstrate that a drug, or presumably an excipient, is not an inhibitor (76). For example, vitamin E TPGS was shown to not inhibit human PEPT1 (55).

Meanwhile, *in vitro* assays showed the BCS Class III drug cimetidine to be a P-gp substrate and inhibitor (9,79,80). This situation exemplifies the general challenge in addressing the question about whether or not excipients in a formulation have potential effects on drug absorption. It is well appreciated that, even for perpetrator drug substances much less perpetrator excipients, that *in vitro* tools to predict *in vivo* transporter impact or transporter-mediated drug interactions have limitations. These limitations include relevance of *in vitro* studies to *in vivo* impact, as well as specificity to one transporter over another transporter, as transporters such as P-gp and BCRP can have overlapping activities. Of note, M9 guidance lists four model drugs for permeability assay method validation: digoxin, paclitaxel, quinidine, and vinblastine. Of these, only digoxin and quinidine are listed as example probes in the FDA clinical drug-drug interaction guidance (77). This lack of convergence reflects that *in vitro* and *in vivo* tools, as well as overall understanding about transporter-mediated interactions at the level of the gut, are still often only modestly developed.

Such limitations are highlighted in examining the current, state-of-the-art recommendations for conducting *in vivo* clinical studies to assess transporter-mediated drug interactions, even for perpetrator excipients (77). This guidance notes clinical substrates of P-gp to include dabigatran etexilate, digoxin, and fexofenadine. The guidance further notes that criteria for selecting P-gp clinical substrates are (a) AUC fold-increase ≥2 with verapamil or quinidine co-administration and (b) *in vitro* transport by P-gp expression systems, but not extensively metabolized. More importantly for one with an interest in assessing excipient risk to modulate P-gp (e.g., risk of excipient to increase drug absorption *via* P-gp inhibition), the guidance notes that criteria for selecting P-gp clinical inhibitor are (a) AUC fold-increase of digoxin ≥2 with co-administration and (b) *in vitro* inhibitor. That is, it is clear that digoxin is the state-of-the-art *in vivo* victim drug to assess a P-gp-mediated drug interaction by a potential perpetrator drug (or excipient). Trueck *et al.* employed digoxin as the P-gp probe in a five-drug cocktail that aims to serve as a clinical tool to screen for transporter-based interactions (81).

However, there are specificity and sensitivity limitations in using digoxin as the P-gp probe for *in vivo* clinical studies (82). Oral absorption of digoxin from tablets is 60–80% (83), reflecting its intestinal permeability is less than high (84). However, with only 20–40% incomplete permeation due to perhaps P-gp, there is a modest amount that P-gp inhibition can increase digoxin absorption. Fexofenadine and dabigatran etexilate have been suggested to be more appropriate P-gp probes than digoxin to assess intestinal P-gp inhibition, although they also have significant limitations (85). Limitations in the availability of a suitable P-gp probe for *in vivo* clinical studies generally reflect the experimental challenges in assessing whether or not excipients in a formulation have potential *in vivo* effects on drug absorption. In other words, transporter effects are difficult to demonstrate or characterize, particularly *in vivo*.

Future research should be aimed to help better answer the critical question — “Are there excipients in the formulation with known or suspected effects on drug absorption?” Currently, *in vitro* and *in vivo* tools as well our overall understanding about transporter-mediated interactions at the level of the gut are often only modestly developed, making this critical question difficult to fully address. This difficulty is further challenged if there is the presumption of an excipient effect, which M9 appears to assume for even BCS Class I drugs.

## CONCLUSION

Excipients affecting GI drug absorption limit the granting of BCS-based biowaivers. Excipients may impact small intestinal transit, passive permeability, or active transport for BCS Class III drugs. BCS Class I drugs are not likely to be impacted by common excipients. However, experience to date supports that common excipients in solid oral IR dosage formulations generally do not modify *in vivo* drug permeability or transit. A few potentially critical absorption-modifying excipients have been identified at high quantities *in vitro* and preclinically, including excipients that can impact intestinal transit (e.g., sorbitol, mannitol). Nonetheless, the current FDA M9 guideline has conservative limits for excipient changes. These restrictions, especially for that of BCS Class III drugs, merit regulatory relief. A database of failed BE clinical trials because of excipient changes could help identify disallowable excipient changes to dosage formulations due to impacting performance.

## Abbreviations

AME: Absorption-modifying excipient
ANDA: Abbreviated New Drug Application
AOT: Sodium 1,4-bis (2ethylhexoxy)-1,4-dioxobutane-2-sulfonate
API: Active drug ingredient
AUC: Area under the plasma-concentration time curve
BCRP: Breast cancer resistance protein
BCS: Biopharmaceutics Classification System
BE: Bioequivalence
Caco-2: Human colon adenocarcinoma cell line
CAS: Chemical abstracts service
*C*_max_: Maximum plasma concentration
CTAB: Cetyltrimethylammonium bromide
EDTA: Ethylenediamine tetraacetic acid
EMA: European Medicines Agency
FDA: Food and Drug Administration
FIP: International Pharmaceutical Federation
GI: Gastrointestinal
HEK293: Human embryonic kidney 293 cells
HPMC: Hypromellose
HT29: Human adenocarcinoma colorectal cell line
ICH: International Council for Harmonisation of Technical Requirements for Pharmaceuticals for Human Use
IID: Inactive Ingredient Database
IR: Immediate release
MDCK: Madin-Darby canine kidney
MDE: Maximum daily exposure
MDR1: Multi-drug resistance mutation 1
MTX: Methotrexate
NaCMC: Sodium carboxymethylcellulose
NDA: New Drug Application
PEG: Polyethylene glycol
PEPT: Proton-coupled oligopeptide transporter
P-gp: P-glycoprotein
PK: Pharmacokinetic
SLS: Sodium lauryl sulfate
SNAC: Salcaprozate sodium
SUPAC: Scale-up and post-approval changes
UNI: Unique ingredient identifier
Vit-E-PEG: D-α-tocopherol poly-(ethylene glycol) succinate
WHO: World Health Organization
